# Supplementary material for: Reactivation during sleep with incomplete reminder cues rather than complete ones stabilizes long-term memory in humans
Source: Commun Biol. 2020 Dec 4;3:733. doi: 10.1038/s42003-020-01457-4 (PMC7718244; doi:10.1038/s42003-020-01457-4)
Supplement: Supplementary file 2 — Description of Additional Supplementary Files [file 42003_2020_1457_MOESM2_ESM.pdf]

Description of Additional Supplementary Files

File Name: Supplementary Data 1

Description: Source data of Study 1. Number of correct responses at training, testing, interference and memory change for each participant included in Study 1 (Experiment 1). Ratings of the Stanford Sleepiness Scale for training, interference and testing for each participant.

File Name: Supplementary Data 2

Description: Source Data of Study 2. Number of correct responses at training, testing, interference and memory change for each participant included in Study 2 (Experiments 2-4). Ratings of the Stanford Sleepiness Scale for training, interference and testing for each participant. Values of the Heard/not-heard task of each participant. Percentage of time in wake, sleep stage 1, 2, slow wave sleep (SWS), rapid eye movement sleep (REM), total sleep time (TST) in minutes, power of slow oscillations (uV^2), delta (uV^2) and spindles (uV^2) for each participant in Experiments 2-4.
