# Supplementary material for: Reactivation during sleep with incomplete reminder cues rather than complete ones stabilizes long-term memory in humans
Source: Commun Biol. 2020 Dec 4;3:733. doi: 10.1038/s42003-020-01457-4 (PMC7718244; doi:10.1038/s42003-020-01457-4)
Supplement: Supplementary file 5 — Reporting Summary [file 42003_2020_1457_MOESM5_ESM.pdf]

nature research

Corresponding author(s): FORCATO CECILIA

Last updated by author(s): Oct 23, 2020

Reporting Summary

Nature Research wishes to improve the reproducibility of the work that we publish. This form provides structure for consistency and transparency in reporting. For further information on Nature Research policies, see Authors & Referees and the Editorial Policy Checklist .

Statistics

For all statistical analyses, confirm that the following items are present in the figure legend, table legend, main text, or Methods section.

n/a	Confirmed	
☐	☒	The exact sample size (*n*) for each experimental group/condition, given as a discrete number and unit of measurement
☐	☒	A statement on whether measurements were taken from distinct samples or whether the same sample was measured repeatedly
☐	☒	The statistical test(s) used AND whether they are one- or two-sided<br>*Only common tests should be described solely by name; describe more complex techniques in the Methods section.*
☒	☐	A description of all covariates tested
☐	☒	A description of any assumptions or corrections, such as tests of normality and adjustment for multiple comparisons
☐	☒	A full description of the statistical parameters including central tendency (e.g. means) or other basic estimates (e.g. regression coefficient) AND variation (e.g. standard deviation) or associated estimates of uncertainty (e.g. confidence intervals)
☐	☒	For null hypothesis testing, the test statistic (e.g. *F*, *t*, *r*) with confidence intervals, effect sizes, degrees of freedom and *P* value noted<br>*Give P values as exact values whenever suitable.*
☒	☐	For Bayesian analysis, information on the choice of priors and Markov chain Monte Carlo settings
☒	☐	For hierarchical and complex designs, identification of the appropriate level for tests and full reporting of outcomes
☐	☒	Estimates of effect sizes (e.g. Cohen's *d*, Pearson's *r*), indicating how they were calculated

Our web collection on statistics for biologists contains articles on many of the points above.

Software and code

Policy information about availability of computer code

Data collection	BrainVision Analyzer (Brain Products)<br>Matlab 2017a (MathWorks Inc, Natick, Massachusetts, USA)
Data analysis	Matlab 2017a (MathWorks Inc, Natick, Massachusetts, USA)<br>MATLAB 2013b (Mathworks, Natick, USA<br>SPSS (version 22.0)<br>open-source toolbox FieldTrip (http://fieldtriptoolbox.org, RRID:SCR_004849)<br>SpiSOP toolbox (https://www.spisop.org, RRID:SCR_015673)

For manuscripts utilizing custom algorithms or software that are central to the research but not yet described in published literature, software must be made available to editors/reviewers. We strongly encourage code deposition in a community repository (e.g. GitHub). See the Nature Research guidelines for submitting code & software for further information.

Data

Policy information about availability of data

All manuscripts must include a data availability statement. This statement should provide the following information, where applicable:

- Accession codes, unique identifiers, or web links for publicly available datasets
- A list of figures that have associated raw data
- A description of any restrictions on data availability

Raw data of study 1 and 2 are included in Supporting Data 1 and 2, respectively. The authors declare that data supporting the findings of this study are available from the corresponding author upon request.

Field-specific reporting

Please select the one below that is the best fit for your research. If you are not sure, read the appropriate sections before making your selection.

☒ Life sciences ☐ Behavioural & social sciences ☐ Ecological, evolutionary & environmental sciences

For a reference copy of the document with all sections, see nature.com/documents/nr-reporting-summary-flat.pdf

Life sciences study design

All studies must disclose on these points even when the disclosure is negative.

Sample size	Sample size was determined based on power analyses with expected effect sizes estimated from previous experiments from our groups.
Data exclusions	We excluded participants from the analysis because of the following reasons: technical problems, difficulties to fall asleep, waking up during reactivation during sleep, not completing all experimental sessions, or snoring-induced repetitive EEG arousals or because they did not reach the learning criterion. These are all indicated in the manuscript.
Replication	We did not explicitly replicate our findings. However, Study 1 can be considered an indirect replication of previous findings from our group.
Randomization	Participants were randomly asigned to each group.
Blinding	Participants were randomly assigned to each group, however, the experimenter was aware of the group assignment because he/she had to administer the correct reactivation protocol during wakefulness or sleep.

Reporting for specific materials, systems and methods

We require information from authors about some types of materials, experimental systems and methods used in many studies. Here, indicate whether each material, system or method listed is relevant to your study. If you are not sure if a list item applies to your research, read the appropriate section before selecting a response.

Materials & experimental systems

n/a	Involved in the study	
☒	☐	Antibodies
☒	☐	Eukaryotic cell lines
☒	☐	Palaeontology
☒	☐	Animals and other organisms
☐	☒	Human research participants
☒	☐	Clinical data

Methods

n/a	Involved in the study	
☒	☐	ChIP-seq
☒	☐	Flow cytometry
☒	☐	MRI-based neuroimaging

Human research participants

Policy information about studies involving human research participants

Population characteristics	None of the participants reported being sick during the experiment, to have any mood disorder or any anxiety disorder, any history of neurological diseases, did not take any medication at the time of the experiments, did not suffer from any sleep disorders,had not been on night shift and did not have any major sleep disturbances during the 6 weeks prior to the experiments. Female participants had a regular menstrual cycle according to self-reports and did not take oral contraceptives. The experiment was scheduled to take place in the luteal phase of the menstrual cycle for all women (15th to 28th day), based on previous evidence that sleep effects for memory are more readily observed in this phase.
Recruitment	Participants were recruited by advertisement at the University of Tübingen.
Ethics oversight	Ethics committee of the Medical Faculty of the University of Tübingen.

Note that full information on the approval of the study protocol must also be provided in the manuscript.
